# Biliary stenting alone versus biliary stenting combined with ^125^I particles intracavitary irradiation for the treatment of advanced cholangiocarcinoma

**DOI:** 10.1038/s41598-019-47791-4

**Published:** 2019-08-05

**Authors:** Qing Pang, Lei Zhou, Xiao-Si Hu, Yong Wang, Zhong-Ran Man, Song Yang, Wei Wang, Zhen Qian, Hao Jin, Hui-Chun Liu

**Affiliations:** Department of Hepatobiliary Surgery, the First Affiliated Hospital of Bengbu Medical College, 233000 Bengbu, Anhui China

**Keywords:** Bile duct cancer, Radiotherapy, Surgical oncology

## Abstract

We aimed to compare the efficacy of percutaneous transhepatic biliary stenting (PTBS) and PTBS combined with ^125^I particles implantation in the treatment of advanced extrahepatic cholangiocarcinoma (EHC). A total of 184 advanced EHC patients, who received PTBS (PTBS group) or PTBS combined with ^125^I particles implantation (PTBS + ^125^I group) from January 2012 to April 2017 in our department, were retrospectively reviewed. The improvement of jaundice and liver function was observed in both groups. The postoperative complications, risk of biliary re-obstruction, and overall survival (OS) were compared between the two groups. Amongst, 71 cases received PTBS and 113 had the additional implantation of ^125^I particles. The jaundice and liver function were significantly improved in all patients, especially in PTBS + ^125^I group. There was no significant difference in the risk of postoperative complications between the two groups. However, the risk of biliary re-obstruction significantly reduced in PTBS + ^125^I group (19.5% *vs*. 35.2%, *p* = 0.017). Kaplan Meier analysis showed that patients in PTBS + ^125^I group had a significantly better OS, both for hilar and distal cholangiocarcinoma. Univariate analysis demonstrated that preoperative levels of carbohydrate antigen 19-9 (CA19-9), total bilirubin, neutrophil count, lymphocyte count, and different therapeutic method were significant factors affecting OS. Multivariate analysis further identified the treatment of PTBS combined with ^125^I particles implantation as an independent protective prognostic factor (HR = 0.26, 95% CI: 0.17–0.39, *p* < 0.001). In conclusion, for patients with advanced EHC, PTBS combined with ^125^I particles implantation is superior to PTBS alone in improving liver function, inhibiting biliary re-obstruction, and prolonging survival time.

## Introduction

Cholangiocarcinoma (CCA) is an epithelial cell malignancy with features of cholangiocyte differentiation. The incidence of CCA has increased globally over the past few decades^1^. The most contemporary classification based on anatomic location includes intrahepatic cholangiocarcinoma (IHC) and extrahepatic cholangiocarcinoma (EHC). Notably, the two types do not only differ in anatomical origin, but also in clinicopathological characteristics. Of the two types, EHC, which consists of hilar cholangiocarcinoma (HCCA) and distal cholangiocarcinoma (DCCA), contributes to 90% of all CCA cases^2^.

Because of the diagnostic difficulty and limited treatment options, the prognosis of EHC is unsatisfactory, with a 5-year survival rate of less than 20%^3^. Currently, surgical resection and liver transplantation are primary radical treatments for early-stage EHC^4,5^. However, the majority of patients with EHC are already at the advanced stage and lose the opportunity of surgery at the time of diagnosis^6^. Chemotherapy with gemcitabine and cisplatin is the standard treatment for inoperable EHC, but the efficacy is poor^6,7^. Radiotherapy is also considered as an effective local modality for unresectable EHC^8^. Biliary drainage and biliary stent implantation have been gradually applied in advanced EHC due to the efficacy of improving biliary obstruction and relieving symptoms. However, because of the rapid tumor development, biliary stenting alone may easily cause the re-obstruction of biliary tract^9^. In contrast, the additional implantation of ^125^I particles can effectively suppress tumor growth through the release of continuous γ rays^10^.

Recent studies have shown that biliary stenting combined with ^125^I particles implantation is a safe and feasible palliative treatment for advanced EHC^11,12^. However, to date, few reports have compared the efficacy of biliary stenting alone with additional implantation of ^125^I particles. Here, we summarized 184 cases of advanced EHC who underwent percutaneous transhepatic biliary stenting (PTBS) or PTBS combined with ^125^I particles implantation in our department. The efficacy, complications, risk of biliary re-obstruction, and overall survival (OS) were compared between the two groups.

## Methods

### Patients

Advanced EHC patients who were admitted to our department from January 2012 to April 2017 were retrospectively analyzed. Inclusion criteria: (1) pathologically or clinically diagnosed as HCCA or DCCA; (2) unresectable or unwilling to surgery; (3) received PTBS (PTBS group) or PTBS combined with ^125^I particles implantation (PTBS + ^125^I group) for the first time. Exclusion criteria: (1) benign biliary stenosis; (2) distant metastasis; (3) previously received surgery, endoscopic stenting, or chemotherapy for EHC; (4) incomplete follow-up data. We reported this study in compliance with STrengthening the Reporting of OBservational studies in Epidemiology (STROBE). Our study was complied with Helsinki Declaration^13^, and was approved by the Ethics Committee of the First Affiliated Hospital of Bengbu Medical College. All patients signed informed consent prior to treatment.

### Surgical procedures

Percutaneous transhepatic cholangial drainage (PTCD) was performed in advance for patients who had obstructive jaundice or biliary tract infection. About one week later, PTBS with or without ^125^I particles implantation were performed under digital subtraction angiography. The process has been described previously^14,15^. The dose delivered by ^125^I particles implantation was approximately at 1 cm from the center of the radioactive source.

### Data collection and follow-up

The electronic medical records were reviewed to collect the following information: patient’s age, gender, tumor location, and preoperative serological examinations, including total bilirubin (TBIL), direct bilirubin (DBIL), alanine aminotransferase (ALT), aspartate aminotransferase (AST), alkaline phosphatase (ALP), gamma-glutamyl transpeptidase (GGT), albumin (ALB), c-reactive protein (CRP), neutrophil count (NC), lymphocyte count (LC), platelet count (PLT), prothrombin time-international normalized ratio (PT-INR), carbohydrate antigen 19-9 (CA19-9), carcinoembryonic antigen (CEA).

All patients were followed-up until September 2017 or until the death. The follow-up contents included biochemical routine, tumor markers, abdomen ultrasound, and/or CT. Changes of serum ALT, AST, TBIL, DBIL, ALP, and ALB were observed at 1, 3, and 6 months postoperatively. When “P” type tube shifted, appropriate adjustment was performed. As ^125^I particles have almost no anti-tumor effect at about 6 months after operation, re-implantation of new ^125^I particles was performed every 6 months. Discarded ^125^I particles were sent to Nuclear Medicine Center for centralized disposal.

### Statistical analysis

We used SPSS 22.0 software for statistical analysis. Continuous variables with normal distribution were expressed as mean ± standard deviation and differences between groups were compared with t test. Or else, median (min-max) with Wilcoxon test was used. Postoperative changes of liver function were analyzed by using paired t test. The optimal cutoff value of continuous variable is determined by the largest value of Youden Index (sensitivity + specificity −1). The primary outcome was OS, which was assessed by Kaplan Meier curves compared with log-rank test. Variables with *P* value less than 0.05 in the univariate analysis were entered into a multivariate Cox regression model. *P* < 0.05 indicates statistically significant difference.

## Results

### Baseline information

A total of 238 advanced EHC patients were admitted to our department from January 2012 to April 2017. Finally, 184 patients met selection criteria and were included in this study. Baseline data of the 184 patients are shown in Table 1. 44 (62.0%) out of the 71 cases in PTBS group versus 73 (64.6%) out of the 113 cases in PTBS + ^125^I group were pathologically or clinically diagnosed as HCCA. There were no significant differences in gender, age, tumor location, serum levels of CA19-9, CEA, ALB, PT-INR, NC, LC, and CRP between the two groups (all *p* > 0.05). However, patients in the PTBS + ^125^I group had significantly higher preoperative levels of ALT, AST, ALP, GGT, and TBIL (*p* < 0.05). The length of hospital stay was slightly longer in the PTBS + ^125^I group, while the difference was not statistically significant (median: 15 *vs*. 13 days, *p* = 0.058). Moreover, hospital costs were relatively higher in PTBS + ^125^I group compared with PTBS group (median: 36,631 *vs*. 28,821 CNY, *p* < 0.001).

**Table 1:** Basic characteristics of the included patients.

Variables	Overall	PTBS group (n = 71)	PTBS + ^125^I group (n = 113)	*p value*
Gender (male/female)	118/66	43/28	75/38	0.424
Age (y)	68.9 ± 12.3	68.8 ± 14.1	68.9 ± 11.0	0.819
Location (HCCA/DCCA)	117/67	44/27	73/40	0.718
CA19-9 (ng/ml)	650.0 (0.6–2068)	886 (1.1–1967)	509.2 (0.6–2068)	0.358
CEA (ng/ml)	4.4 (0.9–490.5)	4.4 (1.0–490.5)	4.4 (0.9–490.5)	0.510
ALT (U/L)	131 (18–542)	106 (18–542)	149 (22–513)	**0.001**
AST (U/L)	134 (19–793)	98 (19–793)	158 (28–541)	**<0.001**
ALP (U/L)	487 (39–2556)	404 (102–1990)	553 (39–2556)	**0.013**
GGT (U/L)	484(4.1–2528)	406(47–1837)	534 (4.1–2528)	**0.047**
TBIL (µmol/L)	224.7 (5.8–706.2)	158 (5.8–551.5)	249.7 (14.1–706.2)	**0.003**
DBIL (µmol/L)	179.3 (3.0–649.0)	150 (3–424)	201.4 (5–649)	**0.006**
ALB (g/L)	34.3 (23.6–45.3)	34.6 (25.1–43.6)	34.2 (23.6–45.3)	0.669
PT-INR	1.03 (0.63–16.5)	1.1 (0.6–1.6)	1.0 (0.7–16.5)	0.234
NC (10^9^/L)	4.5 (1.6–35.6)	4.8 (1.7–34.8)	4.3 (1.6–35.6)	0.236
LC (10^9^/L)	1.3 (0.3–4.2)	1.2 (0.3–2.8)	1.4(0.4–4.2)	0.152
PLT (10^9^/L)	241 (30–644)	252 (30–644)	237 (54–533)	0.817
CRP (mg/L)	16.4 (0.6–270.0)	16.3 (1.5–270.0)	16.4 (0.6–205.6)	0.332
The length of stay (d)	14 (3–47)	13 (3–47)	15 (4–34)	0.058
Hospital costs (CNY)	34,368 (5,533–64,267)	28,821 (14,442–64,267)	36,631 (5,533–61,928)	**<0.001**
Survival time (m)	9 (1–30)	7 (1–13)	12 (1–30)	**<0.001**

*PTBS* percutaneous transhepatic biliary stenting, *HCCA* hilar cholangiocarcinoma, *DCCA* distal cholangiocarcinoma, *CA19-9* carbohydrate antigen 19-9, *CEA* carcino-embryonic antigen, *ALT* alanine aminotransferase, *AST* aspartate aminotransferase, *ALP* alkaline phosphatase, *GGT* gamma-glutamyl transpeptidase, *TBIL* total bilirubin, *DBIL* direct bilirubin, *ALB* albumin, *PT-INR* prothrombin time-international normalized ratio, *NC* neutrophil count, *LC* lymphocyte count, *PLT* platelet count, *CRP* c-reactive protein.

### Evaluation of clinical efficacy

**Figure 1:**
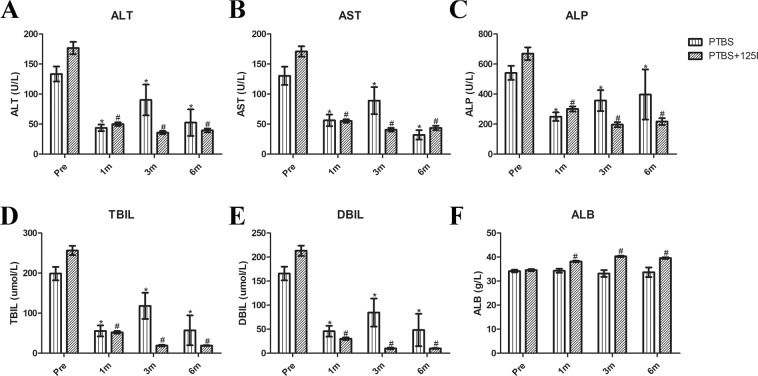
Changes of liver function after operation in the PTBS and PTBS + ^125^I groups. Changes of ALT (**A**), AST (**B**), ALP (**C**), TBIL (**D**), DBIL (**E**), and ALB (**F**) at 1 month, 3 months and 6 months post-operatively (^*^p < 0.05 compared with preoperative values in the PTBS group; ^#^*p* < 0.05 compared with preoperative values in the PTBS + ^125^I group).

All patients had significantly improved clinical symptoms (itching, fever, etc.) in both groups. In the PTBS group, the serum levels of ALT, AST, ALP, TBIL, and DBIL significantly decreased at 1, 3, and 6 months postoperatively, compared with preoperative levels (Fig. 1, all *p* < 0.05). However, no remarkable change of ALB was observed after operation in the PTBS group (*p* > 0.05). In contrast, the changes of all the above serum indices (including ALB) in the PTBS + ^125^I group were more significant than that in the PTBS group.

### Comparisons of complications and risk of biliary re-obstruction

After operation, a total of 9 (12.7%) patients in the PTBS group experienced complications, 6 of which had hyperamylasemia and 3 had biliary tract infection. In contrast, there were complications in 15 (13.3%) patients in the PTBS + ^125^I group. Amongst, 10 cases had hyperamylasemia and 5 had biliary tract infection. No serious complications, radiation-related complications, or perioperative deaths were observed in both groups.

There was no statistically significant difference in overall complications between the two groups. However, compared with PTBS alone, the addition of ^125^I particles reduced the risk of biliary re-obstruction (19.5% *vs*. 35.2%, *p* = 0.017).

### Predictors of OS

**Figure 2:**
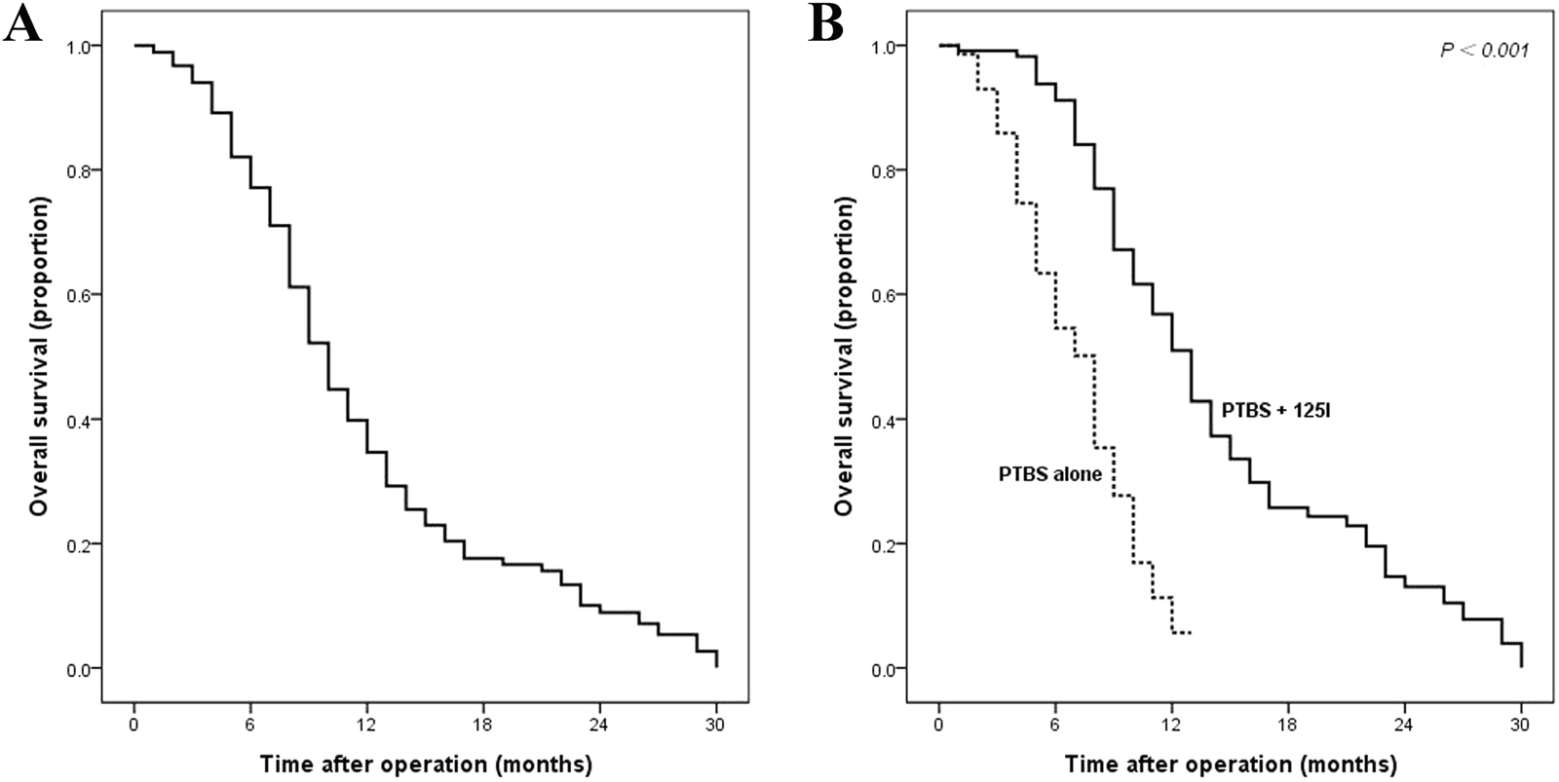
The survival curves of advanced EHC patients. Overall survival curves for the whole cohort (**A**), and stratified according to treatment method (**B**).

During the follow-up period, 150 (81.5%) patients died. As shown in Fig. 2, the OS was significantly better in the PTBS + ^125^I group than that in the PTBS group (median: 13 *vs*. 8 months, 1-year survival rate: 56.8% *vs*. 11.3%, *p* < 0.001).

**Figure 3:**
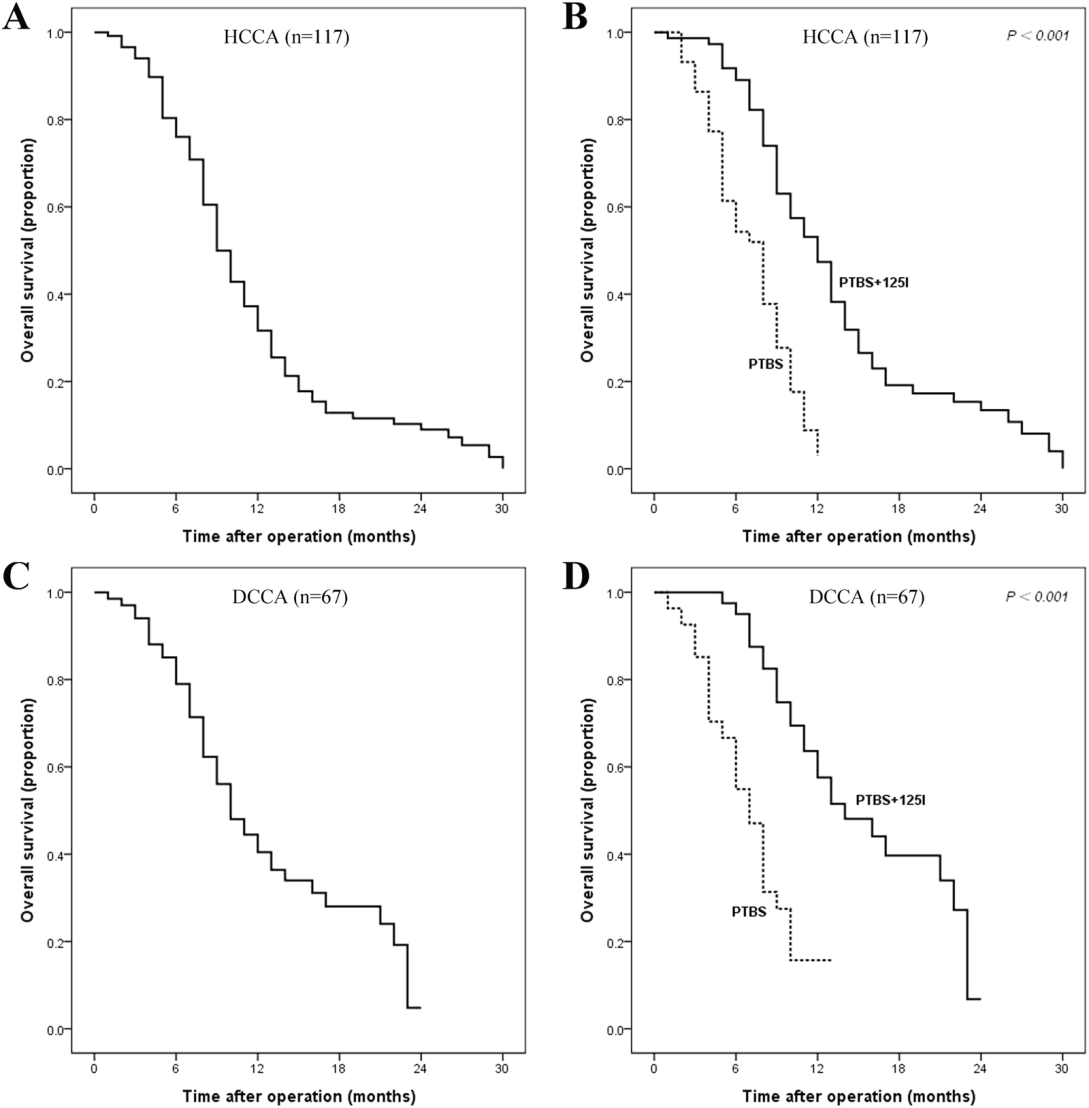
The survival curves of advanced HCCA and DCCA patients. Overall survival curves for HCCA patients (**A**) and stratified according to treatment method in HCCA (**B**); Overall survival curves for DCCA patients (**C**) and stratified according to treatment method in DCCA (**D**).

The comparison of OS was further stratified according to tumor location. The median survival time was 9 months versus 10 months and the one-year survival rate was 37.2% versus 44.5% for patients with advanced HCCA and DCCA, respectively. Compared with PTBS alone, the addition of ^125^I particles can significantly improve OS both in HCCA and in DCCA (Fig. 3, both *p* < 0.001).

**Figure 4:**
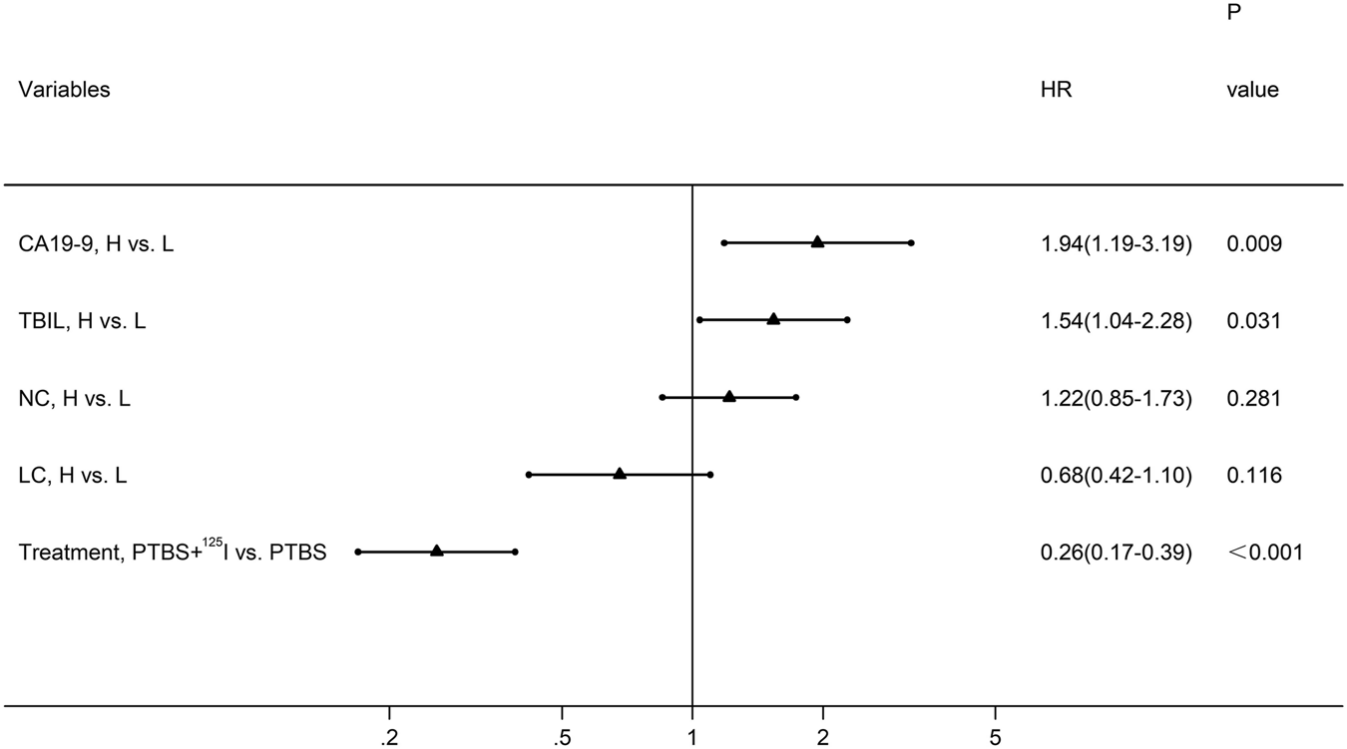
Forest plot based on the results of multivariate analysis of the factors associated with overall survival of EHC patients.

Univariate analysis showed that preoperative levels of CA19-9 (HR = 1.99, 95% CI:1.23–3.23, *p* = 0.005), TBIL (HR = 1.91, 95% CI: 1.33–2.75, *p* < 0.001), NC (HR = 1.44, 95% CI: 1.03–2.00, *p* = 0.032), LC (HR = 0.61, 95% CI: 0.38–0.98, *p* = 0.043), and treatment method (HR = 0.28, 95% CI: 0.19–0.41, *p* < 0.001 were significant prognostic factor affecting OS (Table 2). Subsequently, the above factors were entered into the Cox multivariate regression model. Higher preoperative levels of CA19-9 (HR = 1.94, 95% CI: 1.19–3.19, *p* = 0.009) and TBIL (HR = 1.54, 95% CI: 1.04–2.28, *p* = 0.031) were identified as independent adverse prognostic factors, and the treatment of PTBS plus ^125^I implantation was an independent favorable prognostic factor (HR = 0.26, 95% CI: 0.17–0.39, *p* < 0.001) (Fig. 4).

**Table 2:** Univariate analysis of factors associated with OS of EHC patients.

Variables	Univariate analysis	
	HR (95% CI)	*p value*
Gender (male vs. female)	1.027 (0.736–1.433)	0.876
Age (high vs. low)	1.049 (0.759–1.449)	0.771
Location (DCCA vs. HCCA)	0.826 (0.585–1.168)	0.279
CA19-9 (high vs. low)	1.991 (1.225–3.234)	**0.005**
CEA (high vs. low)	1.131 (0.804–1.591)	0.478
ALT (high vs. low)	1.225 (0.782–1.921)	0.376
AST (high vs. low)	1.188 (0.831–1.697)	0.345
ALP (high vs. low)	1.229 (0.785–1.923)	0.368
GGT (high vs. low)	1.401 (0.791–2.483)	0.248
TBIL (high vs. low)	1.914 (1.334–2.747)	**<0.001**
DBIL (high vs. low)	1.370 (0.976–1.924)	0.069
ALB (high vs. low)	0.764 (0.550–1.063)	0.110
PT-INR (high vs. low)	1.182 (0.826–1.691)	0.360
NC (high vs. low)	1.438 (1.032–2.003)	**0.032**
LC (high vs. low)	0.612 (0.381–0.984)	**0.043**
PLT (high vs. low)	0.878 (0.594–1.298)	0.514
CRP (high vs. low)	1.189 (0.823–1.718)	0.356
PTBS + ^125^I vs. PTBS	0.280 (0.193–0.406)	**<0.001**

*HCCA* hilar cholangiocarcinoma, *DCCA* distal cholangiocarcinoma, *CA19-9* carbohydrate antigen 19-9, *CEA* carcino-embryonic antigen, *ALT* alanine aminotransferase, *AST* aspartate aminotransferase, *ALP* alkaline phosphatase, *GGT* gamma-glutamyl transpeptidase, *TBIL* total bilirubin, *DBIL* direct bilirubin, *ALB* albumin, *PT-INR* prothrombin time-international normalized ratio, *NC* neutrophil count, *LC* lymphocyte count, *PLT* platelet count, *CRP* c-reactive protein, *PTBS* percutaneous transhepatic biliary stenting.

## Discussion

In recent years, the incidence of EHC has increased year by year worldwide. Currently, there are limited treatment options for advanced EHC. It is reported that the 5-year survival rate of advanced EHC is less than 5%^3,16^. Therefore, the exploration of more effective therapeutic methods is critical to improve the outcomes of advanced EHC.

It is generally acknowledged that local progression or metastasis is considered advanced and unresectable for EHC. PTCD can improve the quality of life and has become one of the main treatment for advanced EHC^3^. Compared to palliative surgery, biliary stenting reduces postoperative complications and shortens length of hospital stay^9^. However, due to the rapid growth of tumor, biliary stent alone can easily cause re-obstruction of biliary tract and re-implantation of stent may be needed. In addition, biliary stenting alone shows a limited efficacy in improving survival of EHC^9^.

Radiotherapy is one of the effective treatments for advanced EHC. Recent study has shown that, compared with biliary stenting alone, the addition of external radiotherapy can extend stent patency time (median: 326 *vs*. 196 days, *P* = 0.022) and improve survival time (median: 367 vs. 267 days, *p* = 0.025)^17^. The implantation of radioactive particles is an effective form of brachytherapy^11,18^. Compared with external radiation, the implanted particles can directly act on tumor, and the radioactivity in the tumor is much higher than that in surrounding normal tissues. In addition, brachytherapy can significantly reduce radiation-induced complications such as peptic ulcers, hemorrhage, and radiation enteritis.

Since 2012, PTBS with or without ^125^I particles implantation has been routinely applied for advanced EHC in our department. In this study, we summarized the relevant clinical data of 184 cases with advanced EHC. We found that, compared with PTBS alone, the addition of ^125^I particles significantly reduced the risk of biliary re-obstruction and improved survival, while it did not increase the risk of postoperative complications. No radiation-related complications, such as duodenal ulcer and enteritis, were observed in the PTBS + ^125^I group. It is suggested that, the brachytherapy treatment is safe and may be not limited by some external considerations such as the proximity to the duodenal wall. However, the technique still has several disadvantages. Firstly, as the “P” type tube contained ^125^I particles is implanted in the intra-cavity of biliary stent, ^125^I particles implantation will be performed selectively (usually several days later) provided the stent expansion is insufficient even with balloon assisted dilation. Secondly, the tube contained ^125^I particles may slide or even remove from the stent due to inappropriate postoperative activity.

Multivariate analysis further showed that treatment of PTBS combined with ^125^I particles were an independent favorable factor of OS, and could reduce the risk of death by 74%. Preoperative TBIL and CA19-9 were also identified as important factors affecting the prognosis of EHC, which are consistent with previous reports by Cai *et al*. and Waghray *et al*.^19,20^. However, the effects of postoperative levels as well as dynamic changes of TBIL and CA19-9 in the prognosis of EHC need further research.

The reasons of the addition of ^125^I particles significantly improves outcome in advanced EHC may be manifold. It is reported that ^125^I particles can continuously release X and γ rays to effectively kill tumor cells and inhibit tumorigenesis^10^. Recently, Du *et al*. and Kubo *et al*. showed that, after the implantation of radioactive ^125^I particles, the percentages of CD3 + T, CD4 + T, natural killer (NK), and regulatory T cells significantly increased in peripheral blood of tumor patients^21,22^. In addition, the concentrations of IgM, IgG, and IgA, and complements C3 and C4 also increased, indicating that ^125^I particles may stimulate not only cellular immunity but also humoral immunity^21^.

There are several limitations in the current study. Firstly, because of the relatively small sample size and single-center retrospective design, larger multi-center prospective studies are still required to validate our findings. Secondly, as sampling poses a huge challenge for EHC, the pathological diagnosis is impossible in the majority of cases.

Chemotherapy and radiotherapy are the two major options for advanced EHC. The combination of ^125^I particles brachytherapy with chemotherapy (or chemoradiation) is a promising treatment and the safety and efficacy need to be further studied.

## Conclusions

In conclusion, for patients with advanced EHC, PTBS combined with ^125^I particles implantation is superior to PTBS alone in improving liver function, inhibiting biliary re-obstruction and prolonging survival time.

### Ethical considerations

This study was complied with Helsinki Declaration and was approved by the Ethics Committee of the First Affiliated Hospital of Bengbu Medical College. All patients signed informed consent prior to treatment.

## Data Availability

The raw datasets generated during the current study are available from the corresponding author on reasonable request.
